# Supplementary material for: What role should EBU catheters play in the interventional approach to anomalous right coronary arteries? A case series
Source: PLoS One. 2025 Dec 30;20(12):e0326548. doi: 10.1371/journal.pone.0326548 (PMC12753062; doi:10.1371/journal.pone.0326548)
Supplement: Attachment — Submitted filename: Response to Reviewers.docx [file pone.0326548.s001.docx]

Response to Reviewers

We thank the editors and reviewers for their thoughtful comments and constructive feedback on our manuscript. We have carefully considered all suggestions and have substantially revised the manuscript accordingly. Please find below our point-by-point responses to each comment.Response to Journal Requirements:

Style Requirements: The manuscript has been reformatted to meet PLOS ONE's style requirements.

Data Availability: All underlying data are now available as Supporting Information (S1_Dataset).

Ethics Statement: The ethics statement now appears only in the Methods section.

Tables: All tables are now included within the main manuscript file.

Response to Additional Editor Comments:

Comment: The conclusions were overstated and implied a clinical algorithm.

Response: We have significantly softened the language throughout the manuscript, removing any suggestion of an "algorithm" or "preferred strategy." The conclusions now frame our findings as preliminary insights that support a tailored approach, not a definitive guide. (See Abstract( line 73-75), Introduction(line 110), and Discussion(line 239).

Response to Reviewer #1:
We thank Reviewer #1 for their positive feedback and valuable suggestions.

Comment 1: Expand the limitations section to address selection bias, lack of comparator, and operator experience.

Response: We have expanded the 'Strengths and Limitations' section to explicitly discuss these points, including the selective inclusion of EBU cases and the consequent inability to perform a comparative analysis. (See Limitations section line 256).

Comment 2: Include more commentary on how EBU compares to other catheters in the literature.

Response: We have added a new comparative paragraph in the Discussion that directly compares EBU to Judkins Right, Amplatz Left, and Judkins Left catheters. (See Discussion section line 232-238).

Comment 3: Clarify the composite procedural success in the Results/Table.

Response: We have added a footnote to Table 3 explicitly defining the composite endpoint of procedural success. The results text also now clearly states when success refers to EBU as a first choice versus overall use. (See Table 3 and Results line 190-201).

Comment 4: Correct minor typographical errors.

Response: All noted typos have been corrected throughout the manuscript.

Response to Reviewer #2:
We thank Reviewer #2 for their encouraging words and detailed recommendations.

Comment 1: Add "A Case Series" to the title.

Response: We have amended the title as suggested to: "What Role Should EBU Catheters Play in the Interventional Approach to Anomalous Right Coronary Arteries? A Case Series."

Comment 2: Major English revision.

Response: We have thoroughly polished the manuscript to improve clarity, reduce redundancy, and ensure standard English usage throughout.

Comment 3: Define all abbreviations upon first use.

Response: We have ensured that all abbreviations are defined upon first use.

Comment 4: Clarify anatomical subtypes for second-choice EBU use.

Response: We have clarified this in the Results section, explicitly stating: "one with Type A anatomy, three with Type B anatomy, and one with high take-off anatomy." (See Results line190-192).

Comment 5: Revise in-text citation format.

Response: The citation format has been corrected throughout to use the journal's style (e.g., [1-3]).

Comment 6: Check typo "6(Figure 1)".

Response: The typo has been corrected.

Comment 7: Revise "algorithm" to "strategy" or "framework."

Response: We have replaced "algorithm" with more appropriate terms like "structured description" and "tailored approach" throughout the text.

Comment 8: Define inclusion/exclusion criteria and provide prevalence.

Response: We have clarified the study population in the Methods, stating the total angiogram volume (~31,000), total ARCA patients (68, prevalence 0.22%), and the subset where EBU was used (17). (See Methods line 115-117).

Comment 9: Avoid redundancy in anatomical classification.

Response: We thank the reviewer for this suggestion. We have revised the manuscript to eliminate redundancy. The Introduction now provides a brief conceptual overview of the anatomical classification (line 89-91), while the detailed, operational criteria used for analysis are presented exclusively in the Methods section.

Comment 10: Include tables in the main manuscript.

Response: All tables are now included in the main manuscript file.

Comment 11: Clarify as a case series.

Response: The manuscript is now consistently described as a "case series" in the title and throughout.

Comment 13: Acknowledge selection bias.

Response: We have explicitly acknowledged the selection bias in the Limitations section (line 254).

Additional Statistical Analysis:
As suggested, we have added comprehensive statistical analysis including:

Descriptive statistics with 95% confidence intervals for success rates by anatomical subtype, Fisher's exact tests for categorical comparisons , Kruskal-Wallis tests for continuous variables across subtypes
(See Results section line 191-199)

We believe these revisions have substantially strengthened the manuscript and addressed all concerns raised during the review process. Thank you for considering our revised submission.
